# Assessment of malnutrition in patients undergoing chemotherapy at the National Oncology Centre of the Korle-Bu Teaching Hospital, Accra, Ghana

**DOI:** 10.4314/ahs.v23i4.31

**Published:** 2023-12

**Authors:** Makafui C I Akpah, Olivera Kegey, Kofi Adesi Kyei, Eunice Nortey, Matilda Asante

**Affiliations:** 1 Department of Dietetics, School of Biomedical and Allied Health Sciences, College of Health Sciences, University of Ghana; 2 Department of Radiography, School of Biomedical and Allied Health Sciences, College of Health Sciences, University of Ghana

**Keywords:** Malnutrition, PGSGA Tool, Nutrition assessment, Nutritional status

## Abstract

**Background:**

Globally, cancer is on the rise despite several interventions. The link between nutrition and cancer has long been established with the consequences of poor nutrition on cancer pathway being dire. Early nutrition intervention is recommended for all cancer patients.

**Objective:**

To assess malnutrition among patients undergoing chemotherapy at the National Radiotherapy Oncology and Nuclear Medicine Centre of the Korle-Bu Teaching hospital (KBTH) in Accra, Ghana.

**Methods:**

A cross-sectional study was conducted among 123 patients with different types and stages of cancer who were undergoing chemotherapy. Data was collected from December 2018 to January 2019. The PG-SGA tool was used to assess weight loss at one and six months, food intake and nutrition impact symptoms. A correlation test was used to test the association between PGSGA score and Nutritional triaging. A T-test was used to determine the association between chemotherapy cycles and nutrition. A p-value <0.05 was considered to be significant.

**Results:**

The results revealed that 5.7% (n= 7) of the patients were well nourished, 31.7% (n= 39) were suspected of being malnourished and 62.6% (n=77) were severely malnourished. About half of the participants (48%) had experienced weight loss ranging between 1-20kg with weight loss ≤5kg being most prevalent at both one month and six months prior to the study. More than half (56.9%, n=70) of the participants were consuming less than their usual intake. Majority of the participants had 4-6 nutritional symptoms (39.0%) with symptoms being mostly mild (39.1%). Poor nutritional status was positively correlated with nutritional symptoms (r=0.747, p<0.001).

**Conclusion:**

The PGSGA tool identified that more than half of the patients were severely malnourished hence the need for early nutrition intervention in cancer patients.

## Introduction

Cancer still remains one of the major causes of death worldwide ranking second to cardiovascular diseases 1. Despite several interventions, the prevalence and incidence of cancer keeps increasing worldwide. Globally, it is projected that the number of new cancer cases per year will rise to 23.6 million by 2030 with about 10 and 11 million cancers diagnosed each year in low- and middle-income countries % In 2018, prevalence of cancer and cancer related mortalities in Ghana were estimated to be 42,746 and 15,089 respectively with breast cancer being most prevalent (20.4%) 4.

Indeed, the implication of poor nutrition on cancer onset and progression has long been established % Problems in food intake and overall nutrition are common in cancer management. Globally, malnutrition affects about 15-20% of cancer patients at diagnosis and up to about 80-90% of patients at the advance stages of the disease 7. The PreMiO study demonstrated that malnutrition, anorexia and weight loss were common in cancer patients, even at their first visit to a medical oncology center 8. This prospective, observational study conducted at 22 medical oncology centers across Italy revealed that 51% had nutritional impairment and about 64% had lost weight between 1-10kg 6 months post diagnosis. Malnutrition in cancer is associated with poor treatment outcomes, increased toxicities and reduced survival 8. About 10-20% of death among cancer patients are associated with malnutrition rather than the cancer itself 9. This suggests that the repercussion of poor nutrition on cancer prognosis is huge and should therefore be given a closer attention.

Cancer is a collective term for a large group of distinct diseases characterised by the growth of abnormal cells beyond their normal boundaries that can invade adjoining parts of the body and/or spread to other organs 10. Cancer which can also be called neoplasms and malignant tumours can affect almost any part of the body 10. Malignant tumours can have local or systemic effects that may impact on the nutritional status of the patient. Patients with cancer are at particularly high risk for malnutrition because both the disease and its treatments threaten their nutritional status 11. Studies in hospitals in France and Italy have showed that malnutrition occurs in 30% to 85% cancer patients 12,13. It is estimated that the deaths of 10-20% of cancer patients can be attributed to malnutrition rather than to the malignancy itself 11. Cancer and cancer treatments impact on the nutritional status of cancer patients by altering the metabolic system and decreasing food intake 14. For instance, a tumour may block the digestive tract 15, causing dysphagia, impaired swallowing, early satiety, nausea, vomiting or abdominal pain 16. The tumours or treatment such as chemotherapy can also interfere with gastrointestinal function leading to diarrhoea, constipation and malabsorption 17.

Chemotherapy involves the use of antineoplastic agents or drugs to systemically destroy tumour cells 18-20. Different routes of administering antineoplastic agents include intramuscular, oral, topical, intravenous, intramuscular, subcutaneous and arterial 19. The mode of administration is determined by size, type, position of the tumour and the type of agent to be used and its dosage 19. Chemotherapy is mostly administered in courses and cycles, followed by a recess for three weeks in between cycles which allows healthy cells to regenerate 21.

Chemotherapy does not only act on malignant cells but on other rapidly dividing cells including the mucosa cells of the GIT 21. This may lead to nutritional problems such as altered perceptions of taste and smell, anorexia, nausea and vomiting, food aversions, diarrhoea mucositis, constipation, and early satiety 22. Chemotherapy may also have a direct erosive effect on muscle thereby producing significant loss of body mass 23. Cancer patients often experience weight loss at the time of diagnosis and during chemotherapy 24. Malnutrition in patients undergoing chemotherapy affects the adherence to chemotherapy regimen due to chemo-induced toxicity, which can lead to reduced chemotherapeutic dosage, treatment delays and possibly a definitive termination of treatment 25. It has been established that patients with stabilised weight during chemotherapy have a better progression-free period and overall survival 13. Therefore, it is very important to screen, assess, identify and treat malnutrition early enough.

The Patient Generated Subjective Global Assessment (PG-SGA) is considered the gold standard for nutritional assessment and screening 26. Nutritional assessment of cancer patients undergoing chemotherapy has been carried out in different parts of the world 27-29. In Ghana, however, very little is known about nutritional risk and malnutrition in patients undergoing chemotherapy. This study therefore aimed to use the Patient Generated-Subjective Global Assessment tool to assess malnutrition in patients undergoing chemotherapy at the National Radiotherapy Oncology and Nuclear Medicine Centre of the Korle-Bu Teaching hospital (KBTH) in Accra, Ghana.

## Methods

This cross-sectional study involved 123 cancer out-patients who were undergoing chemotherapy at the National Radiotherapy Oncology and Nuclear Medicine Centre (NRONMC) of the Korle Bu Teaching Hospital, Accra Ghana.

### Study site

The NRONMC was established in 1997 as a clinical department with the Korle-Bu Teaching Hospital. The centre is solely responsible for the management of solid tumours and some benign tumours through the use of ionizing radiation, radio nuclides and chemotherapy. Approximately 1500 patients from various parts of Ghana and the sub region are seen annually. Breast, cervical, prostrate, gastrointestinal tract, head and neck cancers are some of the common cancer cases managed at the centre. Clinic days are Mondays to Fridays with each day allocated for specific cancer groups. Specifically, Monday- Breast Clinic, Tuesday - Gynaecological, Wednesday - Head and Neck, Thursday - Prostate and Central Nervous System and Friday- Gastrointestinal Tract/Sarcoma 30.

### Participants

#### Inclusion criteria

The eligible participants were adults aged 18 years and above who have been diagnosed as having any form of cancer by a physician and undergoing chemotherapy alone or chemotherapy in combination with any other treatment.

#### Exclusion criteria

Patients who were physically weak and unable to talk as a result of their condition and patients who did not give consent to participate were excluded.

### Data collection

The data was collected over a two-month period (December 2018-January 2019). Study participants were recruited during patient review visits to the oncology clinic. Patients on chemotherapy were approached and provided with a thorough explanation of the study. After eligibility criteria applied and consent given, participants were then recruited to the study. Total enumeration sampling method was used in this study

Participants were interviewed to obtain their sociodemographic data: age, gender, educational level, and employment status. Other clinical details such as cancer type, cancer stage, chemotherapy cycles completed, corticosteroids administered and duration of corticosteroid were obtained from their hospital folders.

The PG-SGA tool (v3.22.15), a questionnaire validated for nutritional assessment in oncology patients was used in collecting data 31. A paper version of the PGSGA tool was used to enable the data be transferred into spss for analysis and the digital PGSGA app was used to generate the PGSGA scores. There are two components of the tool: the patient-generated historical items and the professional generated items. The patient-generated historical items include weight, food intake, symptoms and activities and function. The professional generated items include calculation of the percentage weight loss, disease and its nutritional requirements, metabolic demand and physical examination.

### Worksheet 1- Patient-generated historical items including weight, food intake, nutrition impact symptoms and functional capacity

#### Box 1- Weight

Height measurements of participants were taken using a seca stadiometer (model 213 Hamburg, Germany). Participants stood upright on a base plate without shoes with their heads in Frankfurt horizontal plane position and back straight, feet together and heels touching the back of the plate 32. Participants were weighed using a calibrated Full Body Sensor Body Composition Monitor and weighing scale (Omron HB-516C, USA) without their shoes on and no heavy objects in their pockets 32. Weight in the previous one month or six months (if one month is not available) was extracted from participants clinical folders. Participants were asked if they had lost or gained weight in the past two weeks and the answer recorded accordingly.

#### Box 2- Food Intake

The participants were asked to rate their intake of food as compared to their normal intake for the past month stating if it was less than or greater than normal or their usual intake and what they are currently taking. They were then asked their current intake: normal food but less than normal amount, little solid food, only liquids, only nutritional supplements, very little of anything, tube fed or by vein.

#### Box 3 - Symptoms

Nutritional symptoms that have kept participants from eating enough for the past two weeks were assessed. These included vomiting, diarrhoea, dry mouth, things taste funny or have no taste, smells bother me, problems swallowing pain, feel full quickly, fatigue, nausea, constipation, and other causes such as depression, financial or dental problems. Each nutritional symptom has a designated score e.g., nausea has a score of 1 and vomiting has a score of 3. Nutritional symptom scores were generated as sum of the designated scores of the nutritional symptoms stated by the participant e.g., nausea and vomiting would be (nausea (1) and vomiting (3) (1+3=4). the nutritional symptoms scores were grouped as follows; 0-5 indicated low nutrition symptoms score, score 6-10 indicated mild nutrition symptoms score, score 11-15 indicated moderate symptoms score whilst 15-20 indicated severe nutrition symptoms score that will have a negative impact on food intake. Therefore, the higher the nutrition symptom score the more prone you are to poor nutritional status

#### Box 4 - Activities and Function

Performance status or general activity level of the participants over the past month was also enquired.

Worksheet 2 – Disease and its relation to nutritional requirements

The participants' clinical folders were obtained for the type cancer, the stage of disease, age (if >65years) and other relevant diagnosis.

Worksheet 3 – Metabolic Demand

The participants' clinical folders were obtained for intake of corticosteroids and duration, fever and fever duration.

Worksheet 4 – Physical Examination

An assessment (physical examination) of 3 aspects of body composition: muscle status, fat and fluid status were also carried out.

### Data analysis

Unintended weight loss or gain was estimated as the differences between weight at 1 and/ or 6 months prior to study and current weight.

The nutrition information collected was carefully and systematically transferred to the Pt-Global PG-SGA app for analysis. The percentage weight loss was automatically calculated by the Pt-Global PG-SGA app using this formular


$$\frac{\left(one\;or\;six\;months\;weight-current\;weight\right)}{one\;or\;six\;month\;weight}\times 100$$

The patient global app automatically scored both the patient-generated historical items and the professional generated items (PGSGA Score) This score was done by summing up the scores for all the sections (worksheet 1(scoring weight loss), food intake, symptoms, activities and functions, disease and its relation to nutritional requirement, metabolic demand and physical examination). The PGSGA Score was used to determine the nutritional status of the participants. A score from 1-7 was categorised as well nourished (A), a score from 8 to 13 moderate or suspected of being malnourished (B) and a score of 14 and above indicates severe malnutrition (C) 52. The higher the PGSGA score, the poorer the nutritional status. To obtain the triaging recommendations, the scores were further classified according to corresponding nutrition intervention. A score of 0–1 indicates no intervention at this time, 2–3- Patient and family education with pharmacological intervention; 4–8 intervention on symptom management by dietitian The scores were further classified according to corresponding nutrition intervention such that a score of 0–1 indicated regular reassessment and reassurance during treatment; 2–3- Patient and family education with pharmacological intervention; 4–8 intervention on symptom management by dietitian in conjunction with a nurse or physician and a score of >9; indicates a critical need for nutrient support options.^52^,^53^

Statistical analysis was done using the Statistical Package for Social Sciences (SPSS 20.0). Pearson's correlation was used to determine the association between nutrition impact symptoms and the PG-SGA score (nutritional status). An independent T-test was used to determine the association between number of chemotherapy cycles received and nutrition (weight changem nutrition symptoms and PGSGA score). A p-value < 0.05 was considered significant.

### Ethical considerations

This study conformed to the Helsinki Declaration on Human Experimentation of 1975, revised in 1985 and 1989. Ethical approval for the study was obtained from the Korle Bu Teaching Hospital Ethical and Protocol Review Committee – Ethical approval number STC/ IRB/000103/2018. Permission was sought from the National Radiotherapy Oncology and Nuclear Medicine Centre (NRONMC). Informed consent was obtained from all the participants after the study objectives were explained to them.

## Results

Table 1 presents the characteristics of the study participants. Generally, the mean age of the participants was 50.90 ±13.80 years. Majority of the participants were females (79.7%), married (60.2%), educated up to Junior High School level (37.4%) and employed (69.9%). Breast cancer was recorded as the most prevalent diagnosis followed by gynaecological cancers. Other cancers such as Kaposi sarcoma, leiomyosarcoma were the least recorded diagnosis. Almost a third of the participants were at disease stage 3 with a few at stage 1 (6.5%). All participants were on chemotherapy. However about 22% had radiotherapy in addition to the chemotherapy.

**Table 1:** Characteristics of study participants (N=123)

Characteristics	n (%)
Mean age=50.90 ±13.80 years	
*Gender*	
Males	25 (20.3)
Females	98 (79.7)

*Marital status*	
Single	20 (16.3)
Married	74 (60.2)
Separated	15 (12.2)
Widowed	14 (11.4)

*Level of Education*	
Primary	6 (4.9)
Junior High School (JHS)	46 (37.4)
Senior High School (SHS)	21 (17.1)
Tertiary	36 (29.3)
None	14 (11.4)

*Employment Status*	
Employed	86 (69.9)
Unemployed	12 (9.8)
Retired	23 (18.7)
Student	2 (1.6)

*Medical Diagnosis*	
Breast cancer	61 (49.6)
Gynaecological cancers	24 (19.5)
Head and neck cancer	3 (2.4)
Prostate cancer	5 (4.1)
Upper gastrointestinal cancers	7 (5.7)
Osteosarcoma	4 (3.3)
Lung cancer	7 (5.7)
Lower gastrointestinal cancers	8 (6.5)
Others	4 (3.3)

*Stage of Cancer*	8 (6.5)
1	34 (27.6)
2	37 (30.1)
3	36 (29.3)
4	8 (6.5)
Unknown	

*Type of treatment*	
Chemotherapy	97 (78)
Chemotherapy and radiotherapy	26 (22)

*gynaecological cancers: cancer of vulva, uterus, choriocarcinoma, ovary and cervix; other cancers: Kaposis sarcoma, leiomyosarcoma*

### Unintended weight change of participants within 1 or 6 months

**Figure 1:**
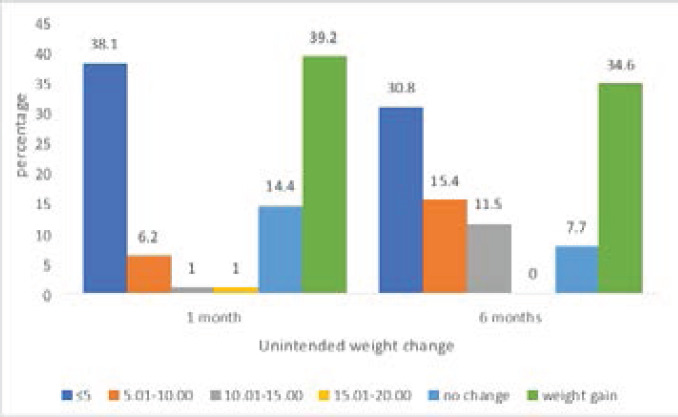
Unintended weight change of participants within 1 or 6 months (N (1month) =97, N (6months) =26)

Weight change of participants within 1 or 6 months are presented in figure 1. About 41.1% and 57.7% of participants had loss weight within 1 or 6 months respectively. Weight loss ranged between 1-20kg with weight loss ≤5kg being most prevalent for both months. Weight loss ≤5kg was more common at 1 month. For participants who had weight at 6 months recorded, weight loss between 5 -15kg seemed more common. There was no weight change among a few participants (14.4% and 7.7%) at both 1 or 6months. Over a third of participants also gained weight within 1 month or 6 months prior to the study.

### Food intake among study participants

**Figure 2:**
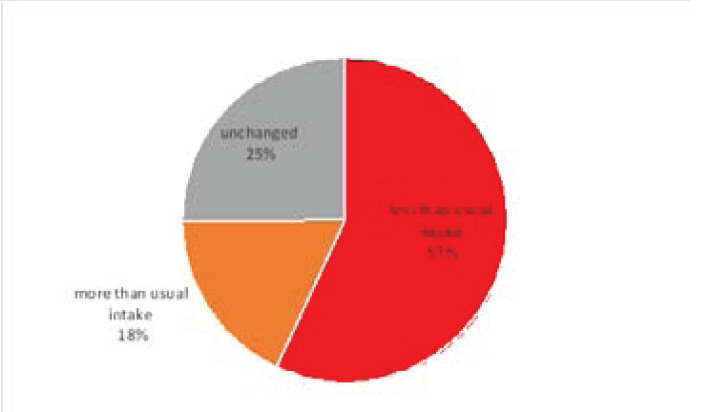
food intake among study participants

Most of the study participants had experienced a change (75%) in their regular food intake with majority of them having less than their usual intake (57%) (figure 2). Close to a third of participants however, recorded no change in their usual food intake (25%).

### Nutritional symptoms among participants

**Figure 3:**
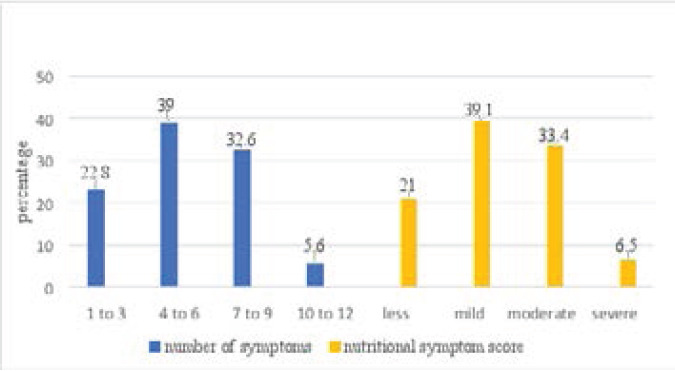
Nutrition symptoms among participants

Nutritional symptoms reported by participants included fatigue, feels full quickly, smells bother me, dry mouth, diarrhoea, vomiting, pain, problems swallowing, things taste funny, mouth sores, constipation, and nausea. Figure 3 shows that majority of the participants had 4-6 nutritional symptoms with symptoms being mostly mild when scored.

### PGSGA scoring and nutritional triaging

**Figure 4:**
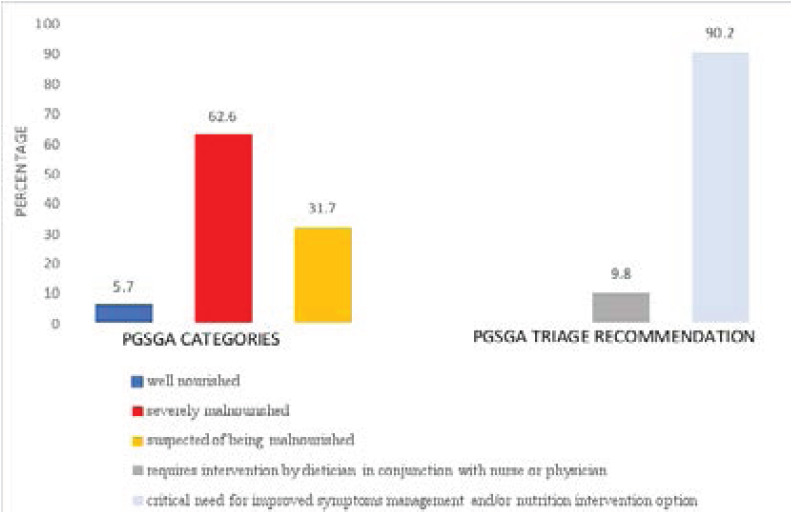
PGSGA scoring and nutritional triaging

PGSGA scoring revealed that over half of the participants (62.60%) were severely malnourished, a third were suspected to be malnourished (31.70%) and about 5.7% were well nourished (figure 4). Nutritional triaging based on the PGSGA score revealed that majority of the participants (90.2%) required the highest level of intervention which indicates a critical need for improved nutritional symptom management and/or nutrient intervention options (figure 3). There was a strong positive significant correlation between the number of nutrition symptoms and the PG-SGA score (r= 0.747, p<0.001).

### Effect of chemotherapy on nutrition

Generally, there seem to be an overall effect of chemotherapy on nutrition of the patients. A T-test revealed that patients who had received less cycles (1- 4 cycles) of chemotherapy had poorer nutrition and were at a higher risk of malnutrition than those who had received more cycles (5-9 cycles) (table 2). These differences were however not statistically significant.

**Table 2:** Effect of chemotherapy on nutrition (T-test)

	Less cycle (1- 4 cycles)	More cycles (5-9 cycles)	P value
Sum of nutritional symptoms	9.26 ± 3.90	7.71 ± 4.17	0.06
% weight loss at 1 month	6.75 ± 7.07	4.66 ± 3.11	0.35
% weight loss at 6 months	9.09 ± 6.10	6.64 ± 4.23	0.27
PGSGA Score	16.46 ± 6.04	15.13 ± 5.43	0.27

## Discussion

This study aimed at conducting nutritional assessment using the Patient Generated-Subjective Global Assessment tool to identify malnutrition in patients undergoing chemotherapy. It is not surprising to have more females than males in this study as two out of the 3 most common cancers in Ghana are seen among females i.e., breast and cervical cancers 4. This was also obvious from this study; breast cancer was most prevalent followed by gynaecological cancers. This study revealed that almost 63% cancer out-patients undergoing chemotherapy at the National Radiotherapy Oncology and Nuclear Medicine Centre (NRONMC) of the Korle Bu Teaching Hospital, Accra were severely malnourished while 31.7% were suspected to be malnourished. Similarly, a systematic review revealed an 83% prevalence of malnutrition in older patients with cancer scheduled to receive chemotherapy 33. Studies in hospitals in France and Italy have showed that malnutrition occurs in 30% to 85% cancer patients 12,13. Malnutrition is common among cancer patients with advanced malignant disease leading to reduced response to treatment, poor prognosis and reduced quality of life 34,35. Indeed, most of the participants in this study were in advance stages hence a possible reason for the high prevalence of malnutrition.

Patients with cancer often develop a continual deterioration of quality of life and nutritional status and this carries prognostic significance 51. It is therefore very important to give much attention to the nutritional status of cancer patients. ESPEN recommends that all cancer patients be screened for malnutrition soon after diagnosis and repeatedly depending on the stability of the clinical situation so as to commence early intervention 14. It is useful to adopt screening protocol that is brief, inexpensive, highly sensitive and have good specificity 14. This can be done via the use of validated screening tools such as Malnutrition Universal Screening Tool (MUST), Malnutrition Screening Tool (MST), Patient Generated Subjective Global Assessment (PG-SGA). This study adopted the use of the PG-SGA tool as it is validated for nutritional assessment in oncology patients 36. This tool also combines both qualitative and semi quantitative data to yield a comprehensive malnutrition score 37. A faster assessment also includes assessing for unintentional weight loss as it has often been reported and recognized to occur frequently in cancer patients and regarded as a stronger and powerful parameter for malnutrition detection among these patients 35. In this current study, almost half of the participants experienced varying degrees of unintentional weight loss. The high prevalence of unintentional weight loss among cancer patients receiving chemotherapy has also been confirmed in other studies with severity directly dependent on the site of the cancer 33,38,39. Hence, cancers located at areas of the body involved with digestion and absorption e.g., lower gastrointestinal cancer are mostly associated with a more severe weight loss (Silva et al., 2015). This narrative is different with the current study possibly because the prevalence of gastrointestinal cancers in Ghana and within the sub-Saharan Region as compared to the other countries is very low 41–43.

Malnutrition is rampant in cancer due to reduced food intake mostly caused by the disease itself or the treatment side effect 14. Cancer and its treatments impact on the nutritional status of the patients by altering the metabolic system and decreasing food intake 14. Specifically, chemotherapy agents cannot distinguish between normal cells and cancer cells. They target cells which are rapidly dividing include healthy ones e.g., bone marrow, hair, GI mucosa, and skin 21. Chemotherapy may cause nutrition related side effects or adverse effects like altered perceptions of taste and smell, anorexia, nausea and vomiting, food aversions, diarrhoea mucositis, constipation, and early satiety 44.

These symptoms have also been confirmed in a number of studies 45–47. Indeed, these nutritional symptoms were also ascertained to be true for this present study. This explains why most of the participants in this study reported experiencing less than their normal dietary intake and ultimately the high prevalence of malnutrition observed in this study. More interestingly, from this study it seemed initial exposures to chemotherapy was associated with worse nutritional outcome compared to more cycles. For instance, patients who had received less cycles had much higher number of nutritional symptoms. This could be because the body recognizes the chemotherapy agents as foreign and reacts at the beginning of the therapy but possibly adjusts with continual exposure. This suggests the need for early nutritional intervention.

Malnutrition or weight loss during chemotherapy is associated with poor treatment outcome because they receive significantly less chemotherapy and develop more toxicity 48. Hence, patients' tolerance to chemotherapy treatment might be affected, due to chemo-induced toxicity, which can lead to reduced chemotherapeutic dosage, treatment delays and possibly a definitive termination of treatment 49,50. It is imperative to screen and treat patients for malnutrition if they are or to prevent it prior to and during treatment. In this study, nutritional triaging based on the PGSGA score supports a critical need of intervention to deal with the nutritional symptoms reported by the patients. The identification, prevention and treatment of malnutrition in cancer patients should be the responsibility of a multidisciplinary team including nurses, physicians, pharmacists, dieticians, and exercise physiologists 14.

Strengths of our study include the use of a validated tool to assess malnutrition and the use of both objective and subjective measurements. This study had limitations such as selection bias however this was mitigated by employing total enumeration method. Also, data was collected on all clinic days until the end of the data collection period. Patients who were too unwell were excluded from the study. The various types of cancers were also not equally represented.

In conclusion, this study revealed a high prevalence of malnutrition in cancer patients undergoing chemotherapy at the National Radiotherapy Oncology and Nuclear Medicine Centre of the Korle Bu Teaching Hospital. Majority of the participants reported experiencing less food intake, weight loss and the occurrence of nutrition impact symptoms such as fatigue, nausea, vomiting etc. These symptoms were positively correlated with the PGSGA score. Ultimately, these findings warrant the need to early nutrition intervention for cancer patients scheduled to undergo chemotherapy.

## Data Availability

Data is available upon request from the corresponding author and reasonable permission from participants of the study.
